# Graph Representation Learning for the Prediction of Medication Usage in the UK Biobank Based on Pharmacogenetic Variants

**DOI:** 10.3390/bioengineering12060595

**Published:** 2025-05-31

**Authors:** Bill Qi, Yannis J. Trakadis

**Affiliations:** 1 Department of Human Genetics, McGill University, Montreal, QC H3A 0C7, Canada; 2 Department of Medical Genetics, McGill University Health Center, Montreal, QC H4A 3J1, Canada

**Keywords:** pharmacogenetics, machine learning, graph representation learning, graph convolutional network

## Abstract

Ineffective treatment and side effects are associated with high burdens for the patient and society. We investigated the application of graph representation learning (GRL) for predicting medication usage based on individual genetic data in the United Kingdom Biobank (UKBB). A graph convolutional network (GCN) was used to integrate interconnected biomedical entities in the form of a knowledge graph as part of a machine learning (ML) prediction model. Data from The Pharmacogenomics Knowledgebase (PharmGKB) was used to construct a biomedical knowledge graph. Individual genetic data (*n* = 485,754) from the UKBB was obtained and preprocessed to match with pharmacogenetic variants in the PharmGKB. Self-reported medication usage labels were obtained from UKBB data field 20003. We hypothesize that pharmacogenetic variants can predict the impact of medications on individuals. We assume that an individual using a medication on a regular basis experiences a net benefit (vs. side-effects) from the medication. ML models were trained to predict medication usage for 264 medications. The GCN model significantly outperformed both a baseline logistic regression model (*p*-value: 1.53 × 10^−9^) and a deep neural network model (*p*-value: 8.68 × 10^−8^). The GCN model also significantly outperformed a GCN model trained using a random graph (GCN-random) (*p*-value: 5.44 × 10^−9^). A consistent trend of medications with higher sample sizes having better performance was observed, and for several medications, a high relative rank of the medication (among multiple medications) was associated with greater than 2-fold higher odds of usage of the medication. In conclusion, a graph-based ML approach could be useful in advancing precision medicine by prioritizing medications that a patient may need based on their genetic data. However, further research is needed to improve the quality and quantity of genetic data and to validate our approach using more reliable medication labels.

## 1. Introduction

To identify the most effective treatment for a patient affected by a given disease, a trial of different candidate medications is often needed. Often, several medications need to be tried before an effective medication is found, causing excessive burden on the patients by prolonging their suffering and decreasing their quality of life. There are also major economic implications. Ineffective treatment and side effects of non-optimal treatment are associated with annual costs of around $495–672 billion in the US (i.e., 16% of total US health care expenditures in 2016) [1].

Pharmacogenomics (PGx) combines pharmacology with genomics with the aim of understanding how genes affect responses to drugs. Genetic associations of medication usage have been identified via genome-wide associations studies. Wu et al. identified 505 linkage disequilibrium independent genetic loci significantly associated with self-reported medication use from 23 medication categories utilizing self-reported medication-use data from the UK Biobank (UKBB) [2]. A more recent meta-analysis of the UKBB, Estonian Biobank, and FinnGen discovered 333 independent genetic loci associated with medication use patterns in hyperlipidemia, hypertension, and type 2 diabetes [3]. These studies, along with the numerous PGx variants and genes identified to be associated with medication response in the literature [4], provides evidence for a role of genetic variations in medication response and usage patterns and a strong case for their use in enabling precision (more targeted) medicine.

Despite the number of significant genetic associations between medication response and usage at the population level, studies leveraging the use of machine learning (ML) methods to predict medication traits at the individual level have been limited. Furthermore, most of the existing studies have been focused on the field of oncology and transcriptomic data [5,6]. However, one recent example from Taliaz et al. have demonstrated the successful use of ML classifiers for predicting the response to specific antidepressants (citalopram, sertraline, and venlafaxine) in major depressive disorder using genetic, clinical and demographic features [7].

Considering the potential of ML analysis of genetic data to enhance the treatment of patients through medication-related predictions, we explored the application of ML to a broader range of medications. The goal of our study is to develop a machine learning (ML)-based model for medication usage prediction based on the genotypes of patients in the UKBB. Our study will address the challenge of reducing the amount of trial-and-error burden on patients by predicting and prioritizing the medications that a patient is likely to use and benefit from. A key assumption underlying our study involves the use of self-reported data on medication usage. Since self-reported medication usage from the UKBB includes only regular medications and health supplements (taken weekly, monthly, etc.), we assume that a patient taking a medication experiences a net benefit (vs. side-effects) from using the medication.

Furthermore, we explored the use of a graph representation learning (GRL) approach to integrate interconnected biomedical entities in the form of a knowledge graph as part of the ML prediction model. There are several successful examples of applications of GRL in the biomedical domain at the molecular, genomic, therapeutic, and healthcare levels [8]. However, to our knowledge, no study has applied a GRL approach for medication usage prediction based on individual genotype data. A biomedical graph capturing the relationships between genetic variants, genes, diseases, and medications could potentially increase the intelligence of ML models and prediction performance by introducing biomedical domain knowledge and dependencies between entities.

## 2. Methods

### 2.1. Graph Convolutional Network

The class of GRL we utilized is the graph convolutional network (GCN) introduced by Kipf & Welling [9]. GCNs extend the ideas of convolution neural networks defined for Euclidean data to irregular graph data. Therefore, we can apply existing neural network operations on graph objects. To illustrate how the GCN works, we first define a graph as consisting of a set of nodes and a set of edges between nodes.

The goal of the GCN is to learn a function (f) of the node features and graph structure. The node features are provided by a feature matrix X with dimensions N×D, where N is the number of nodes in the graph, and D is the number of features per node. The graph structure is represented using an N×N dimensional adjacency matrix A, and an element $A_{ij}$ in the matrix denotes whether an edge exists between nodes i and j.

The function f is a neural network layer consisting of a learnable weight matrix W and a non-linear activation function σ, and takes as input the node feature matrix X and adjacency matrix A:

$$Z=f\left(X,\;A\right)=\sigma ({\hat{D}}^{-\frac{1}{2}}\hat{A}{\hat{D}}^{-\frac{1}{2}}XW)$$

Here, $\hat{A}=A+I$, where I is the identity matrix. $\hat{D}$ is the diagonal node degree matrix of $\hat{A}$. The output of f is a matrix Z with dimensions N×F, where F is the dimensionality of the transformed output. The product of X and W is a transformed node feature matrix, and subsequent multiplication with ${\hat{D}}^{-\frac{1}{2}}\hat{A}{\hat{D}}^{-\frac{1}{2}}$ results in a normalized aggregation of all the transformed neighboring nodes for each node and itself.

The GCN consists of multiple stacked layers of transformations and aggregations for increased expressivity. With $X=H^{\left(l\right)}$, $W=W^{\left(l\right)}$, and $Z=H^{\left(l+1\right)}$, where l is an index denoting the layer of the GCN model, we obtain the layer-wise propagation rule introduced in [9]:

$$H^{\left(l+1\right)}=f\left(H^{\left(l\right)},\;A\right)=\sigma ({\hat{D}}^{-\frac{1}{2}}\hat{A}{\hat{D}}^{-\frac{1}{2}}H^{\left(l\right)}W^{\left(l\right)})$$

The final layer output of the GCN is taken as the embeddings of the nodes in the graph capturing local neighborhood structure around each node. The embeddings can then be used in downstream tasks (e.g., node classification or link-prediction between nodes).

### 2.2. Biomedical Knowledge Graph

The graph data source we utilized for GRL is the Pharmacogenomics Knowledge Base (PharmGKB) [4]. PharmGKB consist of curated relationships between variants, genes, medications, and diseases extracted from PubMed articles using manual curation with the support of natural language processing. The “relationships” table in PharmGKB summarized the curated edges between pairwise entities. From the “relationships” table, we filtered for all edges that have the attribute “associated”, and discarded those that are “ambiguous” or “not associated”, to create a new “filtered relationships” table. Based on the table, a visualization of the graph was generated using the ForceAtlas2 algorithm [10], and partitioned using a community detection algorithm [11], in Gephi (v. 0.10.1) [12]. The visualization of the graph is shown in Supplemental Figure S1.

### 2.3. Individual Genotype Data

For individual genetic data, we used the imputed genotypes from the UKBB (~96 million variants, aligned to + strand of GRCh37 reference genome). We used PLINK2 [13,14] to remove variants with a Hardy–Weinberg equilibrium test *p*-value lower than 1 × 10^−15^, as well as variants with missing call rates exceeding 0.1. We limited our analysis to the autosomes (chromosomes 1 to 22). All variant coordinates were subsequently uplifted to GRCh38, to be consistent with PharmGKB coordinates. Further sample-based filtering was applied based on UKBB recommendations, including the following data-fields: 22010 (recommended genomic analysis exclusions), 22019 (sex chromosome aneuploidy), 22027 (outliers for heterozygosity or missing rate), and 22051 (UKBiLEVE genotype quality control for samples). A list of individuals withdrawn from the UKBB as of 22 February 2022 were removed. After filtering, a total of 485,754 individuals remained.

Variants present in the PharmGKB were matched to those present in the UKBB imputed genotypes dataset. To maximize the number of relevant variants included in the analysis, we matched with variants annotated in the “variants” table in PharmGKB. For variants not part of the “filtered relationships” table, we created additional ad-hoc variant-to-gene and gene-to-variant edges, if the gene corresponding to the variant exists in the “filtered relationships” table. Overall, a total of 3962 variants overlapped between the PharmGKB and UKBB dataset.

Furthermore, we used PGxPOP (https://github.com/PharmGKB/PGxPOP, accessed on 25 April 2025) to obtain inferred haplotype calls based on allele definitions for PGx genes [15]. These haplotypes were matched with the haplotypes present in the “filtered relationships” table. For any haplotypes that were not present in the “filtered relationships” table, we created addition ad-hoc edges linking the haplotype to its corresponding gene. Overall, a total of 175 haplotypes overlapped between the PharmGKB and UKBB dataset.

As a final filtering step to reduce the number of uninformative features for ML analysis, we filtered out any features from the UKBB genotype data with less than 10 non-zero values, resulting in 3890 variant and 156 haplotype features used for subsequent ML analysis.

Supplemental Figure S2 provides a summary of the types of nodes present in the final PharmGKB graph used in our analysis. Supplemental Figure S3 provides a summary of the edge types that exist between node types. The graph is undirected, thus for every edge from node i to j an opposing edge exists from node j to i.

### 2.4. Medication Usage Labels

We obtained medication usage data from UKBB data field 20003 (treatment/medication code). The medication codes are derived from self-reported regular treatments (taken weekly, monthly, etc.). Thus, we assume that an individual taking a medication experiences a net benefit (vs. side-effects) from the medication. To create medication usage labels, we obtained a mapping of UKBB medication codes to ATC codes and active ingredient names from the supplementary data in the study by Wu et al. [2]. Next, for each patient, we converted their UKBB medication codes to ATC codes and active ingredient names. Finally, we filtered for only medications present in the PharmGKB based on a match with either the ATC code or active ingredient name of the medication. To ensure an adequate number of samples for ML analysis, we kept medications taken by at least 100 patients. In total, 264 medications met the threshold. For each patient, a 264-dimensional vector ($y_{i}$) is created with a value of 1 indicating that the patient is taking the medication, and 0 otherwise.

### 2.5. GCN Model Architecture

**Figure 1:**
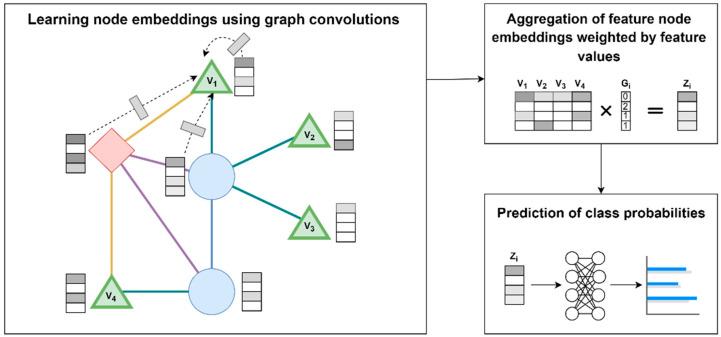
**GCN model architecture.** A simplified illustration of the three steps of the GCN model architecture as described in Section 2.5 is shown. The green nodes represent genetic variants in the graph, while the blue and red nodes are other node types in the graph, such as genes or medications.

A supervised ML approach based on GCN was developed for the prediction of medication usage based on individual genotype data. Of note, the GCN is updated end-to-end jointly with the downstream classifier. Our approach consists of three steps: (1) learning node embedding using graph convolutions, (2) aggregation of feature node embeddings weighted by feature values, (3) prediction of class probabilities. Figure 1 shows an illustration of the approach. In step 1, embeddings for nodes in the graph are learned through graph convolutions, i.e., normalized aggregation of all the transformed neighboring nodes for each node and itself (example shown for node $V_{1}$). The initial features for each node (X) are initialized as an identity matrix. The reasoning for using an uninformative identify matrix is so that the model will leverage only structural information of the graph as part of the predictions, thus emphasizing the benefits of the specific graph structure, and provides a fair comparison against non-graph-based ML approaches. In step 2, the embeddings of nodes corresponding to features (i.e., genetic variant nodes $V_{1}$, $V_{2}$, $V_{3}$, and $V_{4}$), are multiplied with the feature values (i.e., genotypes of a patient i ($G_{i}$)) to yield a feature value weighted aggregation of the embeddings, i.e., patient-specific aggregated embedding ($Z_{i}$). In step 3, $Z_{i}$ is fed into a neural network model to output class probabilities (i.e., predict the medication(s) that patient i is taking).

### 2.6. ML Analysis

We developed supervised ML models using the 3890 variants and 156 haplotypes extracted as described above as features. We performed cross-validation with the data split as 70% training, 10% validation, and 20% testing using a stratified split of individuals to maintain a balanced distribution of each medication across the splits. For cross-validation, we allocated 340,027 individuals for training and 48,576 for validation. A final set of 97,151 individuals was not used in the cross-validation process and was reserved for the final evaluation of the trained models.

To assess the GCN model performance, we compared it with a logistic regression (baseline), a regular deep neural network (DNN), and a GCN model with a randomized graph (GCN-random). All models were trained and evaluated on the same dataset splits. We trained the model using the following multilabel binary cross-entropy loss function with minibatch stochastic gradient descent (SGD) using the Adam optimizer [16]:

$$Loss_{BCE}=-\frac{1}{n}\overset{n}{\underset{i=0}{\sum }}\overset{k}{\underset{j=0}{\sum }}\left(y_{ij}*\log\left({\hat{y}}_{ij}\right)+\left(1-y_{ij}\right)*\log\left(1-{\hat{y}}_{ij}\right)\right)$$

where n is the number of patients in each minibatch for SGD (n = 4096), k is the number of medications (k = 264), $y_{ij}$ is the true label value for patient i and medication j, and ${\hat{y}}_{ij}$ is the model prediction for patient i and medication j.

To select the final model used for evaluation, we ran minibatch SGD until convergence, and selected the model with the lowest $Loss_{BCE}$ value on the validation set. The evaluation metric used to assess and compare model performance is the area under the receiver operating characteristic curve (AUC) on the testing set. An AUC value is calculated for each medication independently. Furthermore, we performed comparisons between approaches using paired (i.e., for each medication) *t*-tests to assess whether the model performance differences are statistically significant. Lastly, we analyzed the mean AUCs across five medication sample size percentile ranges to assess whether there is a relationship between the number of users of a medication and model performance.

### 2.7. Ranking Interpretation of Model Predictions

We performed a downstream analysis assessing the significance of medication usage predictions. Using a ranking approach, we examined whether an individual having a higher rank for a medication (out of all medications) means higher odds of taking the medication. First, for each medication, we standardized the predicted probabilities for each patient into Z-scores with a mean of 0 and standard deviation of 1. Next, for each patient, the Z-scores are converted into ranks (i.e., the higher the Z-score, the higher the rank). For each medication, we use logistic regression to assess the association of having a rank value of the medication ranked in the top five out of all medications with actual medication usage. We limited the analysis to medications with a sample size of at least 5000.

## 3. Results

**Figure 2:**
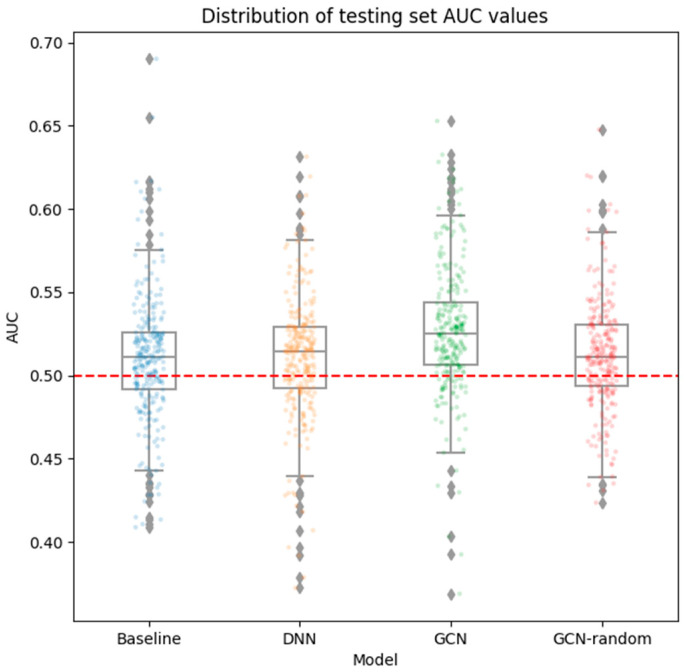
**Distribution of testing set AUC values for each approach.** The performance of each of the final models for each approach is shown. The *y*-axis shows the AUC values while the *x*-axis are labels indicating each approach. The overlayed box-plots show a summary of the AUC ranges, while each point in the strip plot corresponds to the AUC value for a specific medication. The GCN approach achieved the highest mean AUC value over all medications at 0.527, significantly outperforming the Baseline (*p*-value: 1.53 × 10^−9^), DNN (*p*-value: 8.68 × 10^−8^), and GCN-random (*p*-value: 5.44 × 10^−9^) approaches in terms of AUC values.

We selected the models with the lowest $Loss_{BCE}$ value on the validation set for each of the compared approaches (i.e., Baseline, DNN, GCN, and GCN-random). Final evaluations of the selected models were performed on the testing set. The overall mean AUC values on the testing set over all medications for each approach are shown in Table 1. We also provide an overview of the prediction performance over all medications in Figure 2 using a swarm plot of the AUC value of each medication. Overall, the GCN model outperformed the Baseline and DNN models. Using paired *t*-tests, we find statistically significant improvements of the GCN model over baseline (*p*-value: 1.53 × 10^−9^) and DNN (*p*-value: 8.68 × 10^−8^). Furthermore, the GCN model significantly outperforms the GCN-random model (*p*-value: 5.44 × 10^−9^), suggesting that a GCN model utilizing a specific graph structure is significantly better than one with a randomized graph. In summary, the prediction AUCs are statistically better than what would be expected from null predictions; however, in absolute terms, the prediction performance of all approaches was low, ranging from AUC of 0.510 to 0.527.

**Figure 3:**
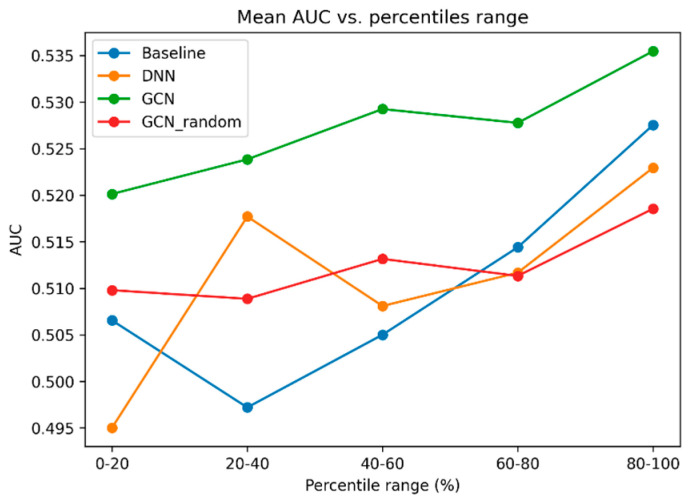
**Mean AUC at each medication sample size percentile range.** The relationship between medication sample size and model prediction performance is shown. The *x*-axis indicates a percentile range of bins of medication sample sizes. Each percentile range is a grouping of medications with sample sizes falling in the range. For example, a percentile range of 0–20 includes all medications that have a sample size that falls within the lowest 20% percentile out of all medications. The *y*-axis indicates the mean AUC of all medications within the percentile range. We noted that as the medication sample size increases, the mean AUC value also increases, suggesting that medications with a higher number of users tend to have better prediction performance. Moreover, the GCN model consistently demonstrates the highest performance across all five percentile ranges.

To explore how model prediction performance is related to the medication sample size (i.e., do medications with a higher number of users have higher AUCs), we plotted the mean AUC value of each bin after binning medications into percentile ranges (Figure 3). Overall, we observe an increase in mean AUC as medication sample size increases. We also observe that the performance of the GCN model is the highest in all five percentile ranges.

**Figure 4:**
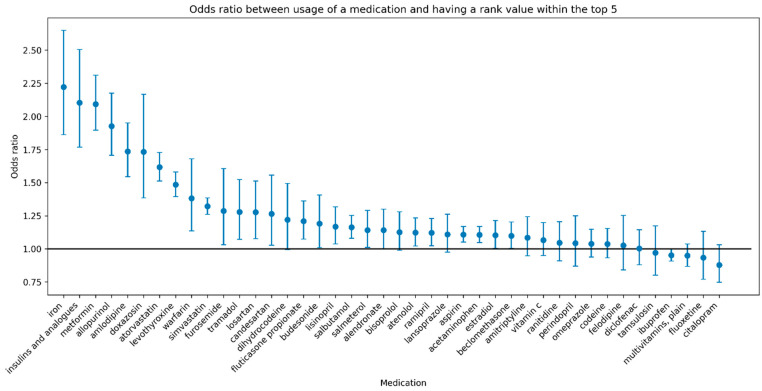
**Odds ratio between usage of a medication and a rank value within the top five.** The odds ratio of the associations between the usage of a medication and having a rank value within the top five is shown for each medication with a sample size of at least 5000. The *x*-axis indicates each medication with a sample size of at least 5000. The *y*-axis indicates the corresponding odds ratio for a rank within the top five for the corresponding medication. The error bars around each point indicate the lower and upper bound of the 95% confidence interval of the odds ratio estimate. Medications are sorted from highest to lowest by odds ratios. We noted that a high rank for several widely used medications, including iron, insulins and analogues, and metformin, is associated with greater than 2-fold higher odds of usage of the medication.

Despite the overall prediction performance in terms of AUC being low, our analysis using the medication ranking approach (described in Section 2.7) revealed a significant association between medication usage and a high rank (a rank in the top five relative to all other medications) for many medications. Figure 4 shows the odds ratio of the associations accompanied by 95% confidence intervals for each medication with a sample size of at least 5000. Furthermore, a high rank for several medications is associated with greater than 2-fold higher odds of usage of the medication (e.g., iron, insulins and analogues, metformin).

## 4. Discussion

Training GCN models on large graphs demands significant computational resources and specialized optimization strategies due to memory constraints, data transfer bottlenecks, and the hybrid nature of sparse-dense operations. In brief, training GCNs on large graphs requires a combination of high-CPU-memory servers, multi-GPU systems, and frameworks that optimize data transfer, parallelism, and memory reuse. Techniques like zero-copy access, hybrid parallelism, and segment training are critical for overcoming scalability challenges.

We introduced a novel application of a graph-based ML approach for medication usage prediction using curated biomedical domain knowledge from the PharmGKB (Supplamental Figures S1–S3) and targeted pharmacogenetic data from 485,754 individuals from the UKBB. Our findings revealed the predictability of 264 commonly used medications based on PGx features (3890 variants and 156 haplotypes). Although the overall performance for medication usage prediction was low, we found that the GCN approach outperformed all other models in our comparison, including the same GCN approach with a random graph, suggesting that the graph structure contained specifically in the PharmGKB graph could be useful to improve the performance of prediction models. Furthermore, using a ranking interpretation of model predictions, we emphasized several medications (iron, insulins and analogues, metformin) that hold significant potential in terms of precision (more targeted) medicine through identifying individuals who are more likely to require these medications.

The relatively poor performance of the models could be due to a combination of different factors. A major limitation of our study is the suboptimal quality of the treatment response labels. Self-reported medication usage is not an optimal measure of therapeutic benefit over side effects. It is not an accurate reflection of response to the medication and introduces significant noise into the analysis due to misclassification. Instead of relying on such a proxy, treatment labels for future studies should be derived from randomized controlled trials (RCTs), or at least be based on electronic health record (EHR) data for efficacy or tolerance (adverse event data). Ideally, N-of-1 clinical trials [17] capturing medication responses and side-effects at the individual level would be required. However, RCTs are expensive to conduct and often have low sample size. As a future direction, it may be feasible to explore transfer learning [18], to leverage the knowledge stored in the current model when training a new model from high quality RCT data.

Another limitation that might have affected the performance of our models is the fact that for the majority of medications, our sample sizes are low. In support of this factor, we saw a consistent trend of medications with higher sample sizes having better performance. At the same time, it should be underlined that filtering medications with >100 users and aggregating by ATC codes may obscure nuances in drug-specific responses, especially for less frequently used or emerging drugs. Furthermore, our input genetic variants are limited in terms of quality of imputation and quantity (in the order of thousands). It may be important to further enrich the current graph to include additional sources of genetic variants and genes with impacts to the metabolism of medications. One potential approach would be to leverage the use of pharmacogenomic variant effect classifiers at the variant level [19], in order to prioritize relevant variants as features for medication trait prediction at the individual patient level.

Despite the relatively poor prediction of individual medications, we found that for several medications, a patient with a higher relative rank of the medication (among all medications) is significantly more likely to be using the medication, thus providing evidence for our method. The ranking interpretation of model predictions could also be more clinically meaningful in the context of precision medicine by providing relative ranks of medications (e.g., for comparing between multiple potential medications).

The medications for which our method had the highest performance include iron, insulins and analogues, and metformin, which are widely used medications. Iron supplementation is used in the treatment of iron deficiency of varying causes including iron deficiency anemia, nutritional deficiency, malabsorption, chronic inflammatory state, blood loss, and others [20]. Insulin is used in the treatment of type 1 diabetes and less commonly in type-2 diabetes by binding to insulin receptors on cells to reduce blood glucose levels [21]. Similarly, metformin is used primarily for the treatment of type-2 diabetes through several mechanisms, including decreasing glucose production in the liver and intestinal absorption, as well as increasing insulin sensitivity [22]. All three medications are associated with greater than 2-fold higher odds of usage of the medication given a rank of the medication within the top five, suggesting that individuals with specific genetic profiles have a higher likelihood of needing these medications. While our method is able to identify individuals requiring iron supplementation, our current method cannot identify individuals who would experience adverse effects of medications due to a lack of ground truth labels for these events. To enable this application in the future, integration of data of medications and adverse events from resources such as electronic health records with natural language processing methods would be required [23]. Lastly, adjustments to the current graph approach, such as enriching the current biomedical knowledge graph with adverse effect nodes and edges to medications, may be needed to enhance the prediction of adverse effects of medications. Finally, although the UKBB is a critical resource, its demographic composition introduces population stratification and participation biases that limit the generalizability of findings to broader populations.

## 5. Conclusions

In conclusion, a graph-based ML analysis of targeted pharmacogenomic variants could be useful in advancing precision medicine by prioritizing medications that a patient may need based on their genetic data. This could help reduce the need for trial-and-error for finding an effective medication. However, further research is needed to validate the findings and explore the clinical utility of our approach. Personalized medicine, particularly when integrated with AI-driven decision-making, introduces significant ethical, regulatory, and clinical challenges. AI’s “black box” nature complicates clinicians’ ability to explain treatment recommendations, potentially eroding patient trust. Algorithmic bias in training data (e.g., underrepresentation in genomic datasets) can disproportionately harm marginalized groups, reinforcing healthcare disparities. Incorrect predictions amplify psychological and clinical harms, demanding robust oversight/regulatory control, patient education, and updated frameworks.

This paper provides preliminary evidence supporting a promising approach. Future studies should focus on improving the quality of treatment labels and genetic data, potentially including whole genome sequencing data to capture all known/unknown informative genetic variants involved in the metabolism of medications. They should also ensure a large sample size for each medication and an independent dataset to validate the generalizability of the model. The most predictive variants/pathways of such a model and the specific graph properties (e.g., centrality, community structure) that contribute to performance improvements should be highlighted.

Future studies should also consider alternative graph-based models, such as GraphSAGE (which is optimized for scalability and inductive learning, using neighborhood sampling and various aggregation functions to efficiently compute node embeddings) and Graph Attention Networks (GAT; leveraging attention mechanisms to assign different importances to neighbors, improving representational power and interpretability).

## Figures and Tables

**Table 1:** Summary of model performance.

Approach	Mean AUC
Baseline	0.510
DNN	0.511
GCN	0.527
GCN-random	0.512

## Data Availability

The genotype and phenotype datasets used in the preparation of this manuscript were accessed from the United Kingdom Biobank (UKBB) through a partnership with the Healthy Brains, Healthy Lives (HBHL) program and Neurohub. The data used to create the graph are available at The Pharmacogenomics Knowledgebase (PharmGKB).

## Supplementary Materials

The following supporting information can be downloaded at: https://www.mdpi.com/article/10.3390/bioengineering12060595/s1, Figure S1: Visual representation of the PharmGKB graph; Figure S2: Node types present in the final PharmGKB graph; Figure S3: Edge types present in the final PharmGKB graph.
