# Supplementary material for: Graph Representation Learning for the Prediction of Medication Usage in the UK Biobank Based on Pharmacogenetic Variants
Source: Bioengineering (Basel). 2025 May 31;12(6):595. doi: 10.3390/bioengineering12060595 (PMC12189576; doi:10.3390/bioengineering12060595)
Supplement: Supplementary file 1 [file bioengineering-12-00595-s001.zip › bioengineering-3637726-supplementary.pdf]

Supplemental Figures

Supplemental Figure S1. Visual representation of the PharmGKB graph.

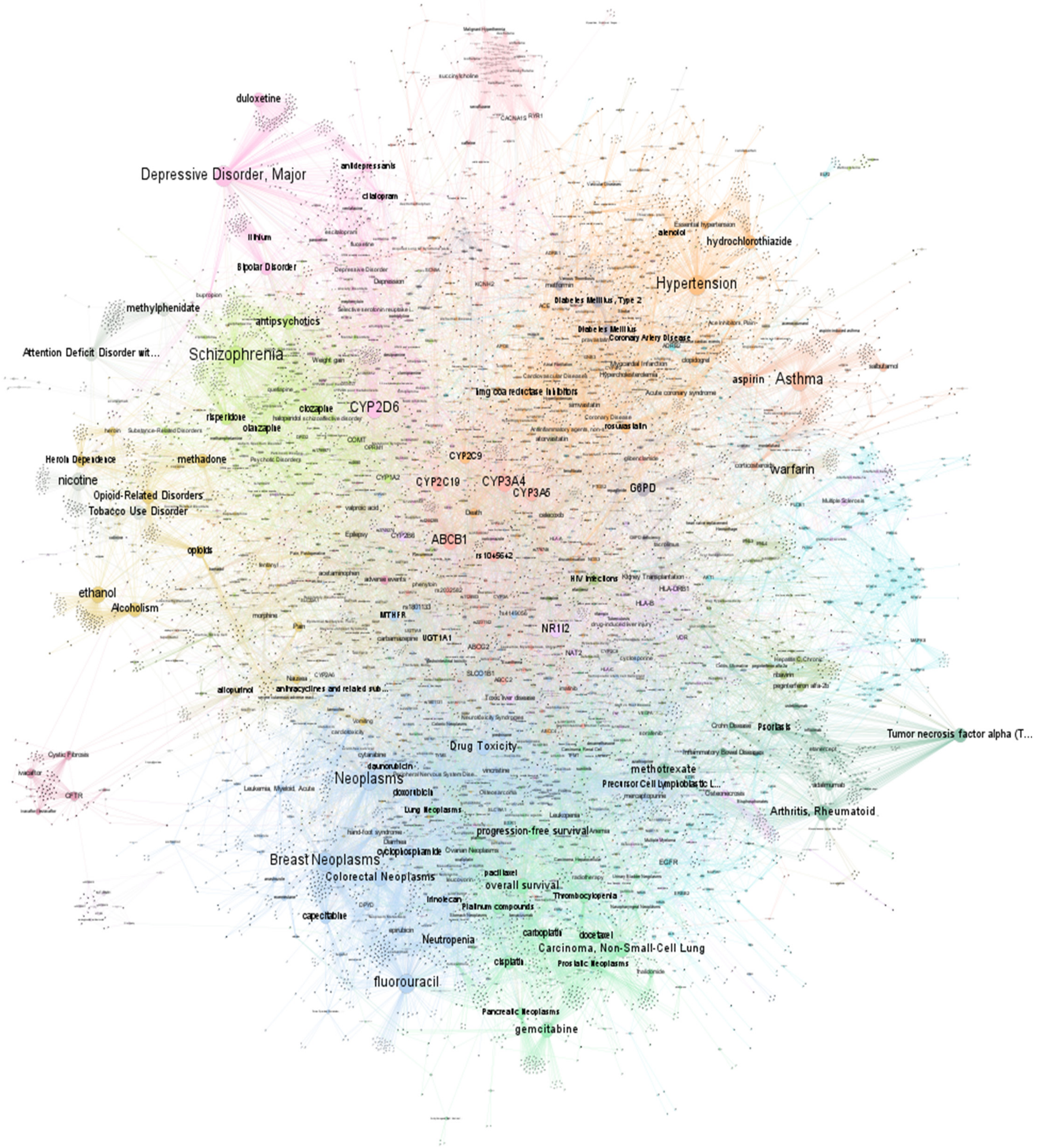

Supplemental Figure S1. A visual representation of the PharmGKB graph is shown. The size of nodes in the graph are defined by their degree (i.e., number of edges a node has). Nodes are colored by the community they belong to as derived from community detection.

Supplemental Figure S2. Node types present in the final PharmGKB graph.

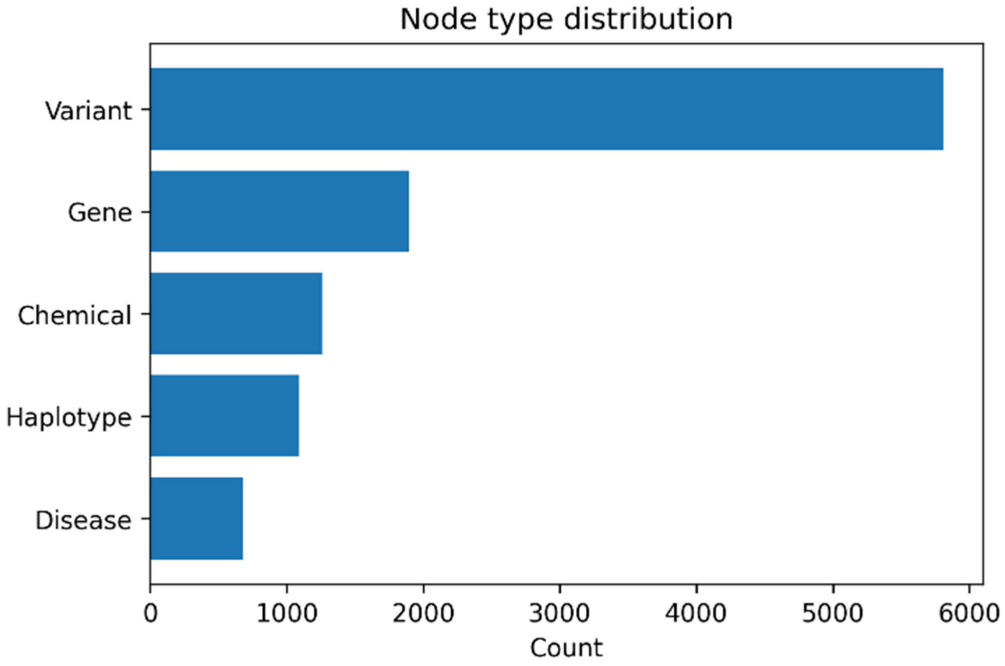

Supplemental Figure S2. The y-axis shows each of the node types present in the final PharmGKB graph, while the x-axis shows the corresponding count. Nodes are sorted by highest to lowest count.

Supplemental Figure S3. Edge types present in the final PharmGKB graph.

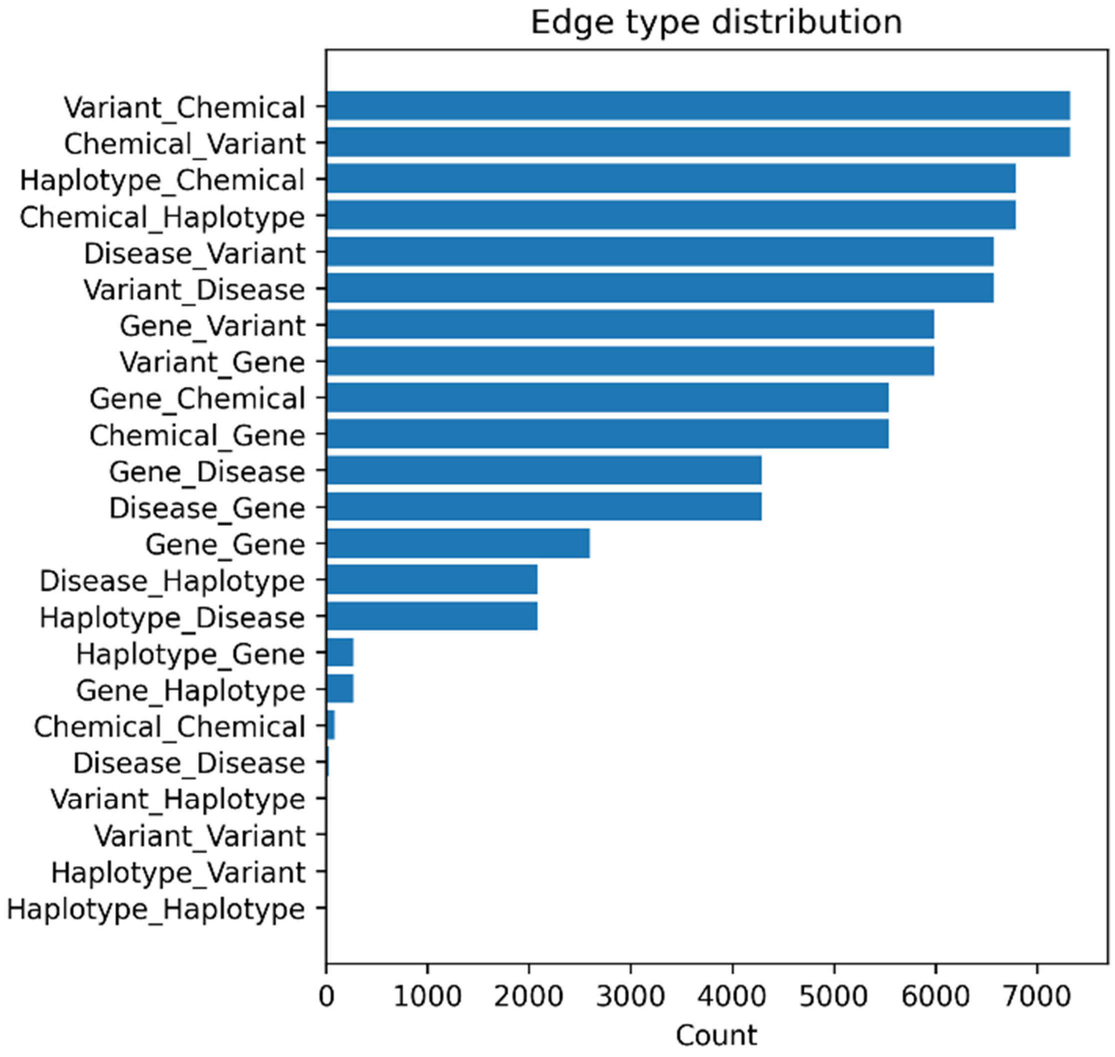

Supplemental Figure S3. The y-axis shows each of the edge types present in the final PharmGKB graph, while the x-axis shows the corresponding count. Edge type labels denote the pair of node types involved in the edge. Since the PharmGKB graph is undirected, an equal number of edges exist for every edge type in the reverse direction. Edge types are sorted by highest to lowest count. Medications are labeled as "Chemical" in the PharmGKB graph.
